# Gut microbiota phospholipids regulate intestinal gene expression and can counteract the effects of antibiotic treatment

**DOI:** 10.21203/rs.3.rs-7924457/v1

**Published:** 2025-12-02

**Authors:** Martin Blaser, Xue-Song Zhang, Meifan Zhang, Yujue Wang, Haipeng Sun, Matthew Scarnati, Zhan Gao, Yue Yin, Christa Zerbe, Emilia Falcone, Mary Lally, Jay Joshi, Sneha Bhattacharya, Maria Diaz-Rubio, Disha Bharj, Disha Patel, Samuel Pan, Gabrielle Ro, Jessica Grenard, Abigail Armstrong, Alexander Valvezan, Steven Holland, Jenifer Mulle, Maria Dominguez-Bello, Xiaoyang Su

**Affiliations:** Rutgers University; Center for Advanced Biotechnology and Medicine, Rutgers University; Center for Advanced Biotechnology and Medicine, Rutgers University; Tsinghua University; Department of Biochemistry and Microbiology, Rutgers University; Center for Advanced Biotechnology and Medicine, Rutgers University; Center for Advanced Biotechnology and Medicine, Rutgers University; Center for Advanced Biotechnology and Medicine, Rutgers University; Clinical Center and NIAID, National Institutes of Health; Clinical Center and NIAID, National Institutes of Health; Center for Advanced Biotechnology and Medicine, Rutgers University; Center for Advanced Biotechnology and Medicine, Rutgers University; Center for Advanced Biotechnology and Medicine, Rutgers University; Metabolomics Shared Resource, Rutgers Cancer Institute; Center for Advanced Biotechnology and Medicine, Rutgers University; Center for Advanced Biotechnology and Medicine, Rutgers University; Center for Advanced Biotechnology and Medicine, Rutgers University; Center for Advanced Biotechnology and Medicine, Rutgers University; Clinical Center and NIAID, National Institutes of Health; Center for Advanced Biotechnology and Medicine, Rutgers University; Center for Advanced Biotechnology and Medicine, Rutgers University; Laboratory of Clinical Immunology and Microbiology, Division of Intramural Research, National Institute of Allergy and Infectious Diseases, National Institutes of Health; Rutgers University; Department of Biochemistry and Microbiology, Rutgers University; Rutgers-Robert Wood Johnson Medical School

## Abstract

Early-life antibiotic exposure perturbs the developing gut microbiome and increases risk for immune disorders such as type 1 diabetes (T1D) in non-obese diabetic (NOD) mice. Comparing germ-free and conventional mice, we identified 747 intestinal lipid compounds and defined a subset of gut microbially-produced lipids (GMPLs). Antibiotic treatment disrupted GMPL profiles in mice and in human volunteers, with partial restoration in mice after cecal microbiota transplantation. Among affected compounds, four phospholipids: LPG(13:0), LPG(16:0), LPG(18:0), and PG(15:0_15:0), were structurally defined and tested functionally. These lipids suppressed LPS-induced NFkB activation, modulated innate immune gene expression in intestinal epithelial cells, and enhanced epithelial cell mitochondrial respiration. Oral administration of LPG(16:0) or LPG(18:0) to antibiotic-treated NOD mice partially restored microbiome composition, normalized ileal gene expression, and improved epithelial transport and metabolic pathways. These findings identify bacterial phospholipids as regulators of intestinal immunity and metabolism, with potential therapeutic applications for inflammatory diseases.

## Introduction

Gut microbes synthesize molecules that influence host metabolism, immunity and neural development^1–3^. Early life is the critical period for microbiome establishment and perturbations during this period can increase risk for immune-mediated diseases^4–8^, including type 1 diabetes (T1D), an autoimmune disease usually beginning in childhood^9–12^. Our previous studies using non-obese diabetic (NOD) mouse models showed that an early-life antibiotic course disrupted the gut microbiota, altered ileal gene expression, and accelerated T1D onset, while cecal microbiota transplant (CMT) partially mitigated these effects^13–15^. To investigate metabolic mediators, we profiled gut microbially-produced intestinal lipids (GMPLs). We then examined how early-life antibiotics and CMT altered GMPLs in NOD mice and tested whether similar effects occurred in humans exposed to antibiotics^16^. These results allowed characterizing specific phospholipids with altered levels in both mice and humans. Evaluating their functional roles *in vitro* and *in vivo,* revealed anti-inflammatory and mitochondrial-stimulating effects, with therapeutic potential for regulating intestinal inflammation.

## Results

**Figure 1:**
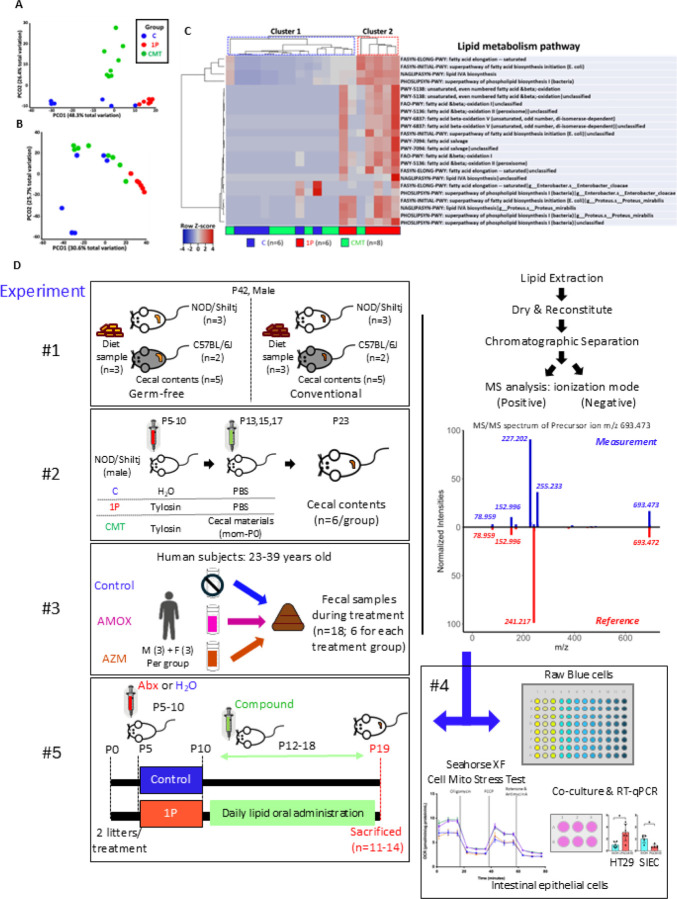
Effect of early-life antibiotic treatment and cecal material transplant (CMT) on fecal bacterial lipid metabolism pathways in mouse feces and subsequent design of lipidomic studies. Analyses based on fecal metagenomic sequencing of P23 male NOD mice in three treatment groups (C, Control; 1P, 1PAT; CMT, CMT following 1PAT). Bray-Curtis distances for pathways identified in the metagenomic analysis with pairwise PERMANOVA analysis: A) 1501 metabolic pathways; B)36 lipid metabolic pathways; C) Heatmap of the 23 identified high-prevalence lipid metabolism pathways. In unsupervised hierarchical clustering, the 1P samples were significantly overrepresented in Cluster 2 (p=0.014; FET). D) Study designs for lipidomic analyses and for administering defined lipid compounds to NOD mice. Experiment 1: Cecal contents of 6-week-old C57BL/6J and NOD/ShiLtJ Germ Free (GF) and conventional mice and their dietary materials. Experiment 2: Cecal contents from male NOD mice at P23 in C, 1P, CMT mouse-groups (n=6/group). Experiment 3: Fecal samples from human volunteers during treatment in the MIME Study, who received one week of (i), azithromycin (AZM), (ii), amoxicillin (AMX), or (iii), no antibiotic (Control). Experiment 4: *in vitro* assays of the defined lipids: with macrophage Raw blue cells to assay expression of key immune regulator NFkB, with intestinal epithelial cells (HT29 colonic cells & mouse small intestinal epithelial cells) to assay immune gene expression, and with HT29 cells to assay mitochondrial respiration. Experiment 5: *in vivo* experiments to assess restoration by administration of specific lipids after early-life antibiotic exposure. 1PAT-exposed pups were gavaged with specific lipids or solvent only for 7 days. Control mice received solvent only. Male pups were sacrificed at P19 for analyses of gut microbiome and intestinal gene expression.

**Figure 2:**
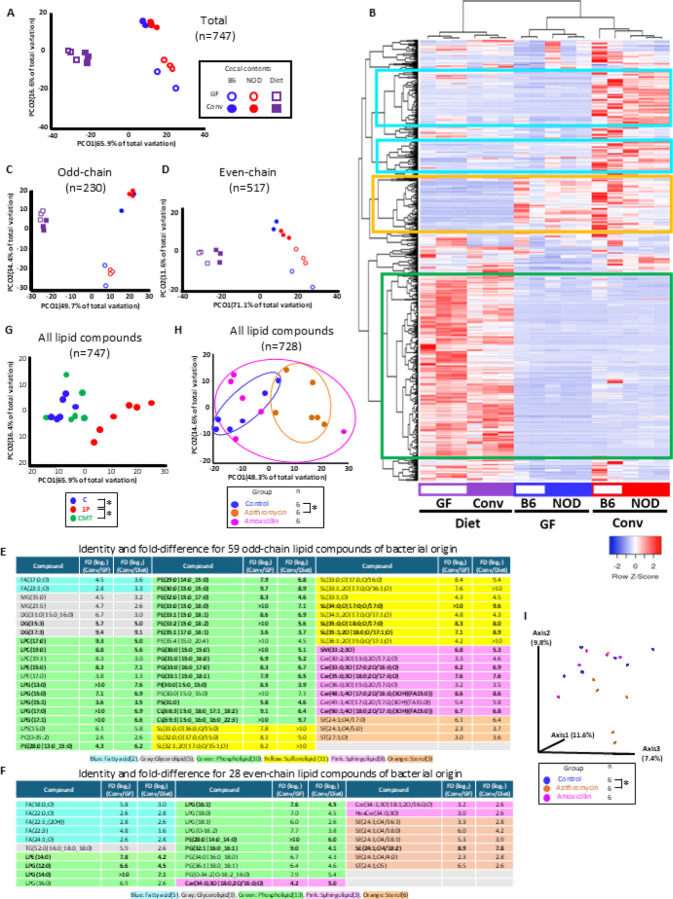
Lipidomic analysis of intestinal contents. Samples were from: 6-week-old Germ-free (GF) and conventional mice and their diets [Experiment 1 (Panels A-F)]: cecal contents of antibiotic-treated NOD mice [Experiment 2 (Panel G)] and antibiotic-treated human volunteers [Experiment 3 (Panels HI)]. A) Bray-Curtis distances of the 747 identified lipids. Differences between conventional vs. GF mice; conventional mice vs. conventional diet; GF mice vs GF diet. Adjusted p values were <0.02 for each comparison, by pairwise PERMANOVA analysis. B) Unsupervised hierarchical clustering based on 747 lipid compounds. Green box: lipids present in the diets metabolized by hosts regardless of microbiota presence or absence. Yellow box: mouse-produced lipids, present in both conventional and GF hosts. Blue boxes: microbially-produced lipids, abundant in conventional cecal contents but not in diets or in cecal contents from GF mice. Bray-Curtis distance matrices (Panels C-D): C) 230 odd-chain; D) 517 even-chain microbially-produced lipid compounds. For both panels, pair-wise PERMANOVA analysis indicated significant differences (adjusted p <0.02) for the same comparisons as in panel A. Identification of 87 significant microbially-produced lipid compounds: E) 59 odd-chain; F) 28 even-chain. Defined by FD(_Conv/GF_) & FD(_Conv/Diet_) >5; compounds meeting both FD-criteria and statistical significance (T-test p<0.05) in bold. G) Bray-Curtis distances for the 747 identified mouse cecal compounds in Experiment 2. Significant differences between C & 1P and between 1P & CMT (adjusted p-value =0.012 for both, pairwise PERMANOVA). H) Bray-Curtis distances for the 728 lipid compounds identified in the Experiment 3 human feces. Pair-wise PERMANOVA analysis indicated significant differences between the control and azithromycin groups (* adjusted p-value=0.015). I) Human fecal p-diversity in Experiment 3: Jaccard distance matrix showing significant intergroup distances (*p=0.02; pairwise PERMANOVA), depth 14,246 reads.

In our prior studies, a single early-life antibiotic course (1P) perturbed the gut microbiota of NOD mice, increasing T1D incidence, while CMT partially restored microbial diversity and reduced risk^15^. Metagenomic analysis revealed 1,501 bacterial metabolic pathways differing among groups, including 36 involved in lipid metabolism, many restored by CMT (Figure 1A–C)^15^. To define the origins of the lipids in intestinal contents, we performed MS/MS lipidomics of diets and cecal contents from germ-free (GF) and conventional mice, identifying 747 non-redundant lipid compounds spanning multiple classes (Tables S1, S2, Figure 1D #1). Both odd-chain lipids (n=230), which are nearly universally of bacterial origin, and even-chain lipids (N=517) from either bacterial and host sources^17^ were identified. Hierarchical clustering identified 349 diet-derived lipids (green), 94 host-produced lipids (yellow), and 144 gut microbially-produced lipids (GMPLs; blue) (Figure 2A–B). Odd-chain GMPLs showed stronger separation than even-chain (Figure 2C–D), and 87 compounds (59 odd-chain, 28 even-chain) met statistical and fold-difference thresholds to be considered as GMPLs, dominated by phospholipids and sphingolipids (Figure 2E–F).

We next examined antibiotic effects on levels of GMPLs (Figure 1D #2) In IP-treated NOD mice, cecal lipid profiles significantly diverged from controls, with partial restoration after CMT (Figure 2G, S3). Clustering revealed distinct groups for control and 1P samples, with CMT samples distributed between them. Of 747 compounds, 343 (45.9%) were decreased by 1P and restored by CMT, and 99 (13.3%) increased by 1P and restored by CMT (Figure S3A). Volcano plot analyses identified 49 significantly altered GMPLs (Figure S3D–F).

Parallel studies in humans tested fecal lipidomes of volunteers receiving azithromycin (AZM, a broad-spectrum macrolide), amoxicillin (AMX, a p-lactam with a narrower spectrum), or no antibiotic (Figure 1D #3). AZM altered microbiota p-diversity (Figure 2I), though a-diversity remained unchanged (Figure S4A). Lipidomics of the human fecal specimens identified 728 compounds, closely overlapping with murine profiles (Table S1). Clustering separated most AZM samples from controls and AMX (Figure 2H, S4B). AZM significantly reduced levels of 31 of 59 odd-chain and 20 of 28 even-chain GMPLs (Figure S4C–D), with strong overlap with mouse data. Phospholipids were the largest antibiotic-depleted class in both host species. However, particular sulfonolipids (SLs) were increased by tylosin in mice but decreased by AZM in humans, highlighting species- and antibiotic-specific responses (Figures S3DF, S4C, S5).

**Figure 3:**
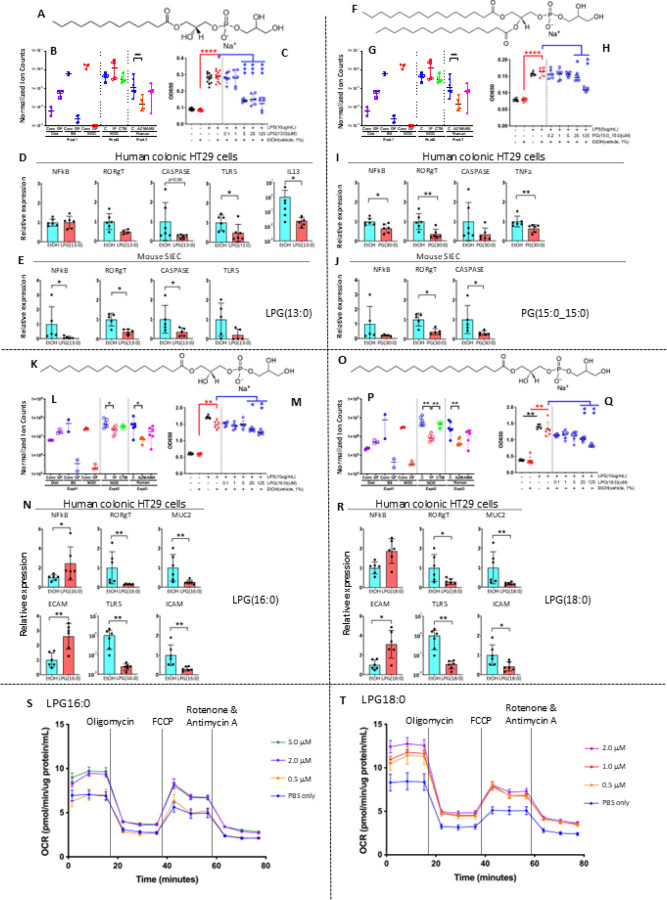
Effects of microbially-produced phospholipids on host immune responses and mitochondrial respiration *in vitro.* Panels A, F, K, O: Chemical structure of LPG(13:0), PG(15:0_15:0), LPG(16:0), and LPG(18:0), respectively. Panels B, G, L, P: Normalized abundances of the four compounds, respectively, in the three experiments (see Figure 2). *p<0.05; **p<0.01; Mann-Whitney CZ-test. Panels C, H, M, Q: Suppressive effects of the four compounds, respectively, on LPS-induced NFkB activity in mouse macrophage Raw Blue cells, respectively. *p<0.05; **p<0.01; ***p<0.001; ****p<0.0001; Mann-Whitney Ltest. Panels D, I, N, R: Effects of the four compounds, respectively, on expression of particular innate immune genes when incubated with human colonic epithelial HT-29 cells. *p<0.05; **p<0.01; Mann-Whitney Latest. Panels E, J: Effects of LPG(13:0) and PG(15:0_15:0) on the expression of innate immune genes in primary small intestinal epithelial cells from male NOD mice at P23. *p<0.05; **p<0.01; Mann-Whitney L-test. Panels S,T: Effects of lipid compounds on mitochondrial respiration and ATP production in HT29 intestinal epithelial cells. HT29 cells were incubated with increasing concentrations of LPG(16:0) (0.5, 2.0, and 5.0 µM) (Panel S) or LPG(18:0) (0.5, 1.0, and 2.0 µM) (Panel T), or with a PBS blank in DMEM medium with 10 mM glucose for 24 h, and mitochondrial respiration assessed using the Seahorse XF Analyzer. Time-course shown of oxygen consumption rate (OCR) of the cells following sequential injection of oligomycin, FCCP, and rotenone/antimycin A. Data represent mean ± SEM; n=7 replicates of each condition.

From GMPLs perturbed in both mice and humans, we focused on four well-defined phospholipids, all phosphatidylglycerols (PGs or lyso-PGs) for structural and functional study: LPG(13:0), LPG(16:0), LPG(18:0), and PG(15:0_15:0) (Figure 3AFKG, Table S3). LPG(16:0) and LPG(18:0) were present in most representative gut colonizing bacterial strains, while LPG(13:0) and PG(15:0_15:0) were absent (Figure S2C, Table S4).

Since we had found that early-life antibiotic exposure accelerated T1D onset by dysregulating genes involved in host intestinal innate immune responses^15^, we investigated whether any of the studied PG/LPGs affected transcription of NFkB, the master regulator of innate immune responses^18^. In functional assays, none of the four compounds altered NFkB activity alone (Figure S6G,J–M). However, each significantly suppressed LPS-induced NFkB activation in macrophages in a dose-dependent manner, paralleling retinoic acid (Figures 3CHMQ, S6H)^18,19^. This suppression was observed across all four PG/LPGs, indicating a shared anti-inflammatory property. In intestinal epithelial cells (HT29) and mouse small intestinal epithelial cells (SIEC), the compounds differentially regulated innate immune genes, variably down-regulating RORyT and CASP1, while may influence NFkB and TLR5 (Figure 3DEIJNR).

Because phospholipids are known to affect energy metabolism through regulating mitochondrial functions^20,21^, we examined mitochondrial activities in HT29 cells. Both LPG(16:0) and LPG(18:0) increased basal and maximal respiration, ATP-linked respiration, and non-mitochondrial respiration, without altering coupling efficiency (Figures 3S–T, S7). Parallel assays in HEK293 cells yielded similar results. Confocal microscopy analysis of HT29 cells stained with MitoTracker and LysoTracker showed that HT29 cells treated with LPG(18:0) had a significantly higher proportion of normal mitochondria (1–5 µm in length) and trending toward fewer elongated mitochondria (5–10 µm). A similar, though nonsignificant, trend was observed in cells treated with LPG(16:0). These data provide evidence that both compounds improve mitochondrial health, decreasing mitochondrial stress without detectable toxicity (Figure S7OP).

**Figure 4:**
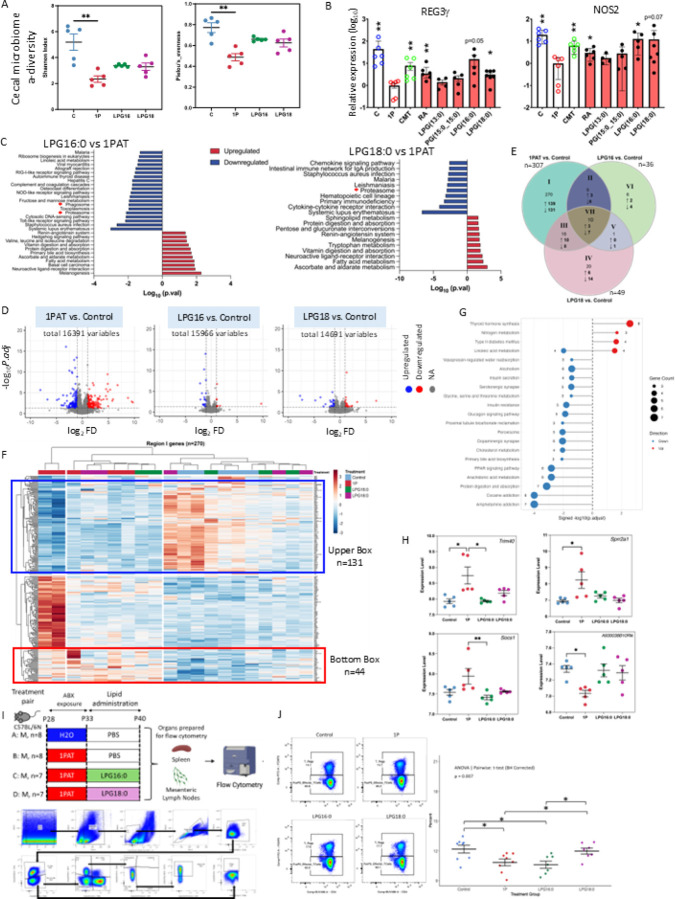
Effects *in vivo* of LPG(16:0) and LPG(18:0) administration after antibiotic treatment. Control NOD mice were compared with those that received an early-life antibiotic course (1P) and then one of the specified phospholipids or not (n=5 mice/group; See Methods for details). A) Cecal microbiome a-diversity (Shannon index and Pielou Evenness) showing the1P significant decrease compared to Control, while administration of LPG(16:0) or LPG(18:0) trend to restore a-diversity. B) RT-qPCR-based mouse ileal expression of T1D early markers (REG3g, NOS2). Mean (± SD) log_10_ relative expression values shown. *p<0.05 compared with 1P group; Mann-Whitney Utest. C) Significantly differentiated KEGG pathways based on RNAseq analysis comparing 1PAT treatment alone or followed by administration of LPG(16:0) (Left) or LPG(18:0) (Right). indicates the two pathways (phagosome and proteasome) downregulated by LPG(16:0) after 1PAT that are closely linked to mitochondrial physiology and function. D) Volcano plots of differential ileal genes in pairwise comparisons (n=5 mice/group): 1PAT vs Control (Left); LPG(16:0) vs Control (Middle); and LPG(18:0) vs Control (Right). E) Venn diagram of the differentially expressed genes (DEGs) in the Control ilea, compared with the antibiotic-treatment (1PAT) alone (1PAT; n=307) or antibiotic treatment followed by administration of LPG(16:0) (n=36), or LPG(18:0) (n=49). Numbers indicate total DEGs for each comparison, with arrows indicating upregulated (T) and downregulated (|) genes, respectively. The relatively small number of DEGs in the LPG(16:0) and LPG(18:0) groups indicates that both treatments partially restored gene expression toward baseline (Control) levels. F) Unsupervised hierarchical clustering of the 270 genes in which 1P was significantly differentiated from Control and restored by LPG(16:0) and LPG(18:0) (Region I in Panel E). G) KEGG pathway enrichment analysis of the 270 Region I genes, identifying pathways disrupted by 1P and restored toward control expression by both LPG(16:0) and LPG(18:0). H) Expression of representative genes modulated by treatment. Trim40, Sprr2a1, and Socs1 were significantly up-regulated by antibiotic (1P) but returned to baseline with either lipid, whereas uncharacterized transcript A930038B10Rik was down-regulated by 1P with partial restoration by both LPG(16:0) and LPG(18:0). Data represent Mean ± SD; significance determined by Mann-Whitney U-test. *p<0.05, **p<0.01. I) Experimental design for spectral flow cytometry analysis of spleen and mesenteric lymph node (MLN) immune cells from C57BL/6N mice exposed to 1PAT and subsequent lipid administration. Male C57BL/6N mice received an antibiotic treatment course(1PAT) from postnatal day 28 to 33, followed by a 7-day administration of PBS (Control = 1P alone), LPG(16:0), or LPG(18:0), and spleens collected for flow cytometry. J) Representative flow cytometry gating strategy (Left) used to identify FoxP3^−^ effector T cells from total splenocytes across the four treatment groups, and quantitation (right) of FoxP^−^ effector cell percentages among total lymphocytes. Data presented as mean ± SEM. Statistical analysis was performed using one–way ANOVA followed by pairwise t-tests with Benjamini-Hochberg (BH) correction. p < 0.05; p < 0.01.

To examine effects *in vivo,* we next gavaged antibiotic-treated NOD pups with LPG(16:0) or LPG(18:0) (Figure 1D #5). Both molecules partially restored cecal and ileal microbiota structure, including Lactobacillaceae g_HT002 (Figures 4A, S8A–D). RNA-seq revealed that antibiotics induced broad ileal transcriptomic shifts, which were partially normalized by LPG treatment (Figures 4BDF, S9AB). KEGG analyses highlighted recovery of epithelial transport and metabolic pathways, while suppressing proteasome and phagosome pathways (Figure 4CG). Of 307 antibiotic-altered genes, 270 were unique to the antibiotic treated mice; in contrast, only 36 and 49 others were found to be altered by LPG(16:0) and LPG(18:0), respectively, indicating restoration toward baseline (Figure 4EFH, Table S5). Notably, genes associated with mitochondrial and lysosomal functions were identified (Table S6).

To assess immunological effects, we evaluated systemic (splenic) and local (mesenteric lymph node, MLN) immune cell populations in C57BL/6N mice exposed to antibiotics without or with added phospholipids using a 26-marker spectral flow cytometry (Figure 4I) panel (Table S7). The 1PAT treatment significantly reduced the proportions of splenic FoxP3^−^ effector T cells, helper T cells, and naïve T cell populations, and administration of LPG(18:0), but not LPG(16:0), significantly restored these populations toward control levels (Figures 4J, S10AB). 1PAT exposure led to significant reductions in multiple CD45^+^CD19^−^ immune cell populations in MLN of the same mice, but without restoration from the subsequent LPG(16:0) or LPG(18:0) treatments (data not shown).

In total, these results demonstrate that early-life antibiotics disrupt intestinal GMPLs in mice and humans, that selected bacterial phospholipids suppress inflammation and enhance mitochondrial activity, and that oral administration of LPG(16:0) or LPG(18:0) partially reverses the antibiotic-induced dysbiosis, and ileal transcriptional and splenic T cell changes.

## Discussion

Our findings identify a distinct set of gut microbially-produced phospholipids (GMPLs) that act as host modulators, counteracting early-life antibiotic disruption in both mice and humans. By systematically comparing lipid profiles in conventional and germ-free mice, we defined odd- and even-chain lipids of microbial origin, then showed that antibiotics markedly reduce these GMPLs, with partial restoration after cecal microbiota transplant (CMT). The overlap between antibiotic-perturbed lipids in NOD mice and in human volunteers underscores the cross-species relevance of these microbial products.

We focused on four phospholipids: LPG(13:0), LPG(16:0), LPG(18:0), and PG(15:0_15:0), that were consistently altered in both antibiotic-treated mice and humans and structurally defined. In functional assays, each suppressed LPS-induced NFkB activation in macrophages, and modulated innate immune gene expression in intestinal epithelial cells. These results directly link bacterial phospholipids to suppression of pro-inflammatory signaling, highlighting a mechanism through which the microbiome may shape immune homeostasis^18,19^.

Beyond immune regulation, these bacterial phospholipids directly enhanced mitochondrial respiration in intestinal epithelial cells. Both LPG(16:0) and LPG(18:0) increased basal and maximal oxygen consumption, ATP-linked respiration, and non-mitochondrial respiration in HT29 cells. These findings indicate that GMPLs contribute not only as structural or metabolic intermediates but also as regulators of epithelial bioenergetics. Because mitochondria supply ATP for epithelial barrier integrity and generate reactive oxygen species that modulate both innate and adaptive immune responses^22–25^, their stimulation by bacterial phospholipids provides a plausible mechanism by which the microbiome sustains epithelial resilience. Given the centrality of mitochondrial function in inflammation and tissue repair^19,26,27^, these findings suggest that bacterial phospholipids influence epithelial resilience via bioenergetic support.

In vivo, gavage of antibiotic-treated NOD mice with LPG(16:0) or LPG(18:0) partially restored the microbiome and normalized ileal gene expression profiles. RNA-seq revealed broad antibiotic-induced transcriptomic shifts, many of which were corrected by LPG administration. KEGG analyses indicated restoration of transport and metabolic pathways and suppression of proteasome and phagosome activity, consistent with reduced inflammatory tone. Restoration of antibiotic-induced depletion of helper T cell populations is another salutary effect. Together, these results demonstrate that specific bacterial LPGs can recapitulate some of the protective effects of CMT, but in a chemically defined form.

The structural diversity of bacterial phospholipids reflects microbial adaptation to complex niches and differentially mediate host-microbe communication^28–31^. Our results show that a subset exert potent, conserved effects on host immunity and metabolism. These findings extend previous observations linking lipid metabolites to immune regulation^26^ and indicate that early-life depletion of GMPLs may contribute to increased risk of immune-mediated diseases such as T1D. By restoring these lipids, either via microbiota transfer or direct supplementation, it may be possible to counteract antibiotic-induced dysbiosis and preserve mucosal immune balance.

In summary, this work defines bacterial phospholipids as mediators of host-microbiome communication. Their dual roles—suppressing NFKB-driven inflammation and enhancing mitochondrial activity-support their potential as therapeutic agents for inflammatory disorders, including T1D and IBD^27^, that originate in the gut.

## Methods

### Lipid compound standards.

SPLASH LipidoMIX Mass Spec Standard, including PC(15:0_18:1-d7), PE(15:0_18:1-d7), PS(15:0_18:1-d7), PG(15:0_18:1-d7), PI(15:0_18:1-d7), PA(15:0_18:1-d7), LPC(18:1-d7), LPE(18:1-d7), cholesteryl ester(18:1-d7), MG(18:1-d7), DG(15:0_18:1-d7), TG(15:0_18:1-d7_15:0), SM(18:1-d7), and cholesterol(d7)] were purchased from Avanti Polar Lipids (cat# 330707, Birmingham, AL) and used as internal standards for lipidomic MS analysis. For use as references for specific cecal lipid MS2 analyses and for both in vitro and in vivo experiments, we purchased Lysophosphatidylglycerols LPG (13:0) [1-tridecanoyl-sn-glycero-3-phospho-(1’-rac-glycerol) (sodium salt)], LPG (16:0) [1-palmitoyl-2-hydroxy-sn-glycero-3-phospho-(1’-rac-glycerol) (sodium salt)], & LPG (18:0) [1-stearoyl-2-hydroxy-sn-glycero-3-phospho-(1’-rac-glycerol) (sodium salt)], and phosphatidylglycerol PG (15:0_15:0) [1,2-dipentadecanoyl-sn-glycero-3-phospho-(1’-rac-glycerol) (sodium salt)] (Avanti Polar Lipids), and retinoic acid (RA) and E *coli* lipopolysaccharides (LPS) was purchased from Sigma-Aldrich (cat# L2143–10MG, Saint Louis MO).

### Preparation of lipids.

Total lipids were extracted using the methyl tert-butyl ether (MTBE) method. Fresh extraction solvent was prepared on the same day of total lipid extraction by mixing 0.1 M hydrochloric acid (Sigma-Aldrich cat# 1090601000), methanol (Sigma-Aldrich cat# 34860), and the SPLASH LipidoMIX Internal Standard in a 500:495:5 v/v/v ratio. For each 10–15 mg sample of cecal contents, feces, or mouse diet, 50 µL fresh extraction solvent was added to 2 mL screw cap tubes containing 200 µL of 0.1 mm glass beads (BioSpec cat# 11079101, Bartlesville OK). The samples were homogenized at 2000 rpm for 45 seconds, then paused for 45 seconds, repeating for 5 cycles to avoid overheating. The mixture was then combined with 2 volumes of MTBE, vortexed for 30 seconds, and left to stand at room temperature for 1 minute to allow layer separation. The top MTBE layer containing total lipids (600 µL) from each sample was collected and air-dried in a fume hood for ~16 hours. The dried samples were stored at −80°C until mass spectrometry analysis performed at the Metabolomics Shared Resource, Rutgers Cancer Institute. The dried total lipid samples were resuspended in 150 µL of resuspension solvent (isopropanol mixed with methanol at 1:1 v/v), followed by centrifugation at 13,000 g for 10 minutes. The top layer (100 µL) was then transferred to glass autosampler tubes.

### Liquid chromatography-mass spectrometry analysis.

Reverse phase separation was performed on a Vanquish Horizon UHPLC system (Thermo Fisher Scientific, Waltham MA) with a Poroshell 120 EC-C18 column (150 mm x2.1 mm, 2.7 pm particle size, Agilent InfinityLab, Santa Clara CA) using a gradient of solvent A (90%:10% water : methanol with 34.2 mM acetic acid, 1 mM ammonium acetate), and solvent B (75%:25% isopropanol : methanol with 34.2 mM acetic acid, 1 mM ammonium acetate). The gradient was 0 min, 25% B; 2 min, 25% B; 5.5 min, 65% B; 12.5 min, 100% B; 19.5 min, 100% B; 20.0 min, 25% B; 30 min, 25% B. The flow rate was 200 µl/min. The injection volume was 5 µL and the column temperature was 55 °C. The autosampler temperature was set to 4°C and the injection volume was 5µL. The full scan mass spectrometry analysis was performed on a Thermo Q Exactive PLUS with a HESI source which was set to a spray voltage of −2.7kV under negative mode and 3.5kV under positive mode. The sheath, auxiliary, and sweep gas flow rates were 40, 10, and 2 (arbitrary unit) respectively. The capillary temperature was set to 300°C and the aux gas heater was 360°C. The S-lens RF level was 45. The m/z scan range was set to 100 to 1200 m/z under both positive and negative ionization modes. The AGC target was set to 1e6 and the maximum IT was 200 ms. The resolution was set to 140,000 at m/z 200. All samples were analyzed by positive and negative electrospray ionization (ESI+/ESI−) in full scan MS mode. Quality control samples (pooled QC) were prepared by combining equivalent volumes of each sample and were used in data-dependent MS-MS (ddMS2) acquisition for lipid identification purposes.

Targeted lipidomics for confirming Iipid identity were performed by Parallel Reaction Monitoring (PRM) acquisition mode. The PRM parameters were as follows: default charge, 1; resolution 17,500 at m/z 200; isolation window, 2 m/z; AGC target was set to 1e5 and the maximum IT was 100 ms. Stepped normalized collision energy (NCE) was set to 20, 30, and 40.

### Lipidomic Data Processing.

Thermo RAW data files were acquired using Xcalibur 4.3 software (Thermo Scientific) and were converted to ABF format using the ABF converter (accessible at: http://www.reifycs.com/AbfConverter). The lipid annotation was performed using MS-DIAL 4.9 (http://prime.psc.riken.jp/compms/msdial/main.html) and the lipid quantitation was performed using El-MAVEN. Normalization was done based on the Splash^®^ Lipidomix^®^ internal standards.

### Mice.

The NOD/ShiLtJ Germ-free mice were obtained from Kathy D. McCoy laboratory of University of Calgary and C57BL/6J Germ-free mice were purchased from Charles River Laboratories (Wilmington DE) and were bred and maintained in a gnotobiotic facility at Rutgers New Jersey Medical School with sterilized chow diets (LabDiet 5LG4, LabDiet, St. Louis MO). Conventional NOD/ShiLtJ mice and C57BL/6J mice were purchased from Jackson Laboratory (Bar Harbor ME), conventional C57BL/6N mice were purchased from Taconic Farms (Germantown NY). Mice were bred or maintained in an SPF vivarium at Rutgers University’s School of Public Health animal facility and at the Robert Wood Johnson Medical School Research Tower animal facility, with chow diets (PicoLab 5058, LabDiet). Both germ-free and conventional mice (NOD/ShiLtJ, C57BL/6J and C57BL/6N) in the above facilities were maintained in the same environment of 23 °C ± 1 °C, on a 12:12-hour light-dark cycle. All animal procedures were approved by the Rutgers University Institutional Animal Care and Use Committee (IACUC protocols no. 201900013, 201900017 and 201900032).

### Human fecal specimens.

Fecal specimens were obtained from young adults (age 23–39 years) enrolled in the Microbial, Immune, and Metabolic Perturbations by Antibiotics (MIME) Study^16^. Subjects received amoxicillin (AMX, 500 mg every 12 hours for 7 days), or azithromycin (AZM, 500 mg on day 1, followed by 250 mg every 24 hours for 4 days), or no antibiotic (Control). Each treatment group had 6 subjects: 3 males/3 females. The fecal samples were collected and directly frozen at −20°C at participants’ homes and then transported in dry ice to study laboratories at NIH, and subsequently to Rutgers University, where they were frozen at −80°C for microbiome and lipidomic analysis. All procedures were approved by the NIH Institutional Review Board (IRB protocol 16-I-0078).

### Microbe strains.

All bacterial strains were grown at 37°C in an anaerobic Chamber (Coy Lab Products, Grass Lake MI) under an atmosphere of 90% N2, 5% CO2, and 5% H2 for 24–72 h. *Alistipes finegoldii* strain 23–018 (isolated from P23 NOD mouse cecal contents in Blaser Lab) was cultured in Brain Heart Infusion (BHI) medium (Thermo Scientific, Waltham MA), *Muribaculaceae spp.* strain MH8C (gift from the Carolina Tropini Lab) and was cultured on Tryptic soy agar (TSA) with 5% sheep blood agar medium (Thermo Scientific), *Escherichia coli*strain Nissle 1917 was cultured in Luria-Bertani (LB) broth medium, *Bifidobacterium longum* strain HMXZ001 (isolated from ECAM baby feces in Blaser lab) was cultured in Bifidobacterium Selective Medium (BSM) (Millipore Sigma, Burlington MA), *Clostridium perfringens* strain S107 (ATCC, Manassas VA) were cultured in Reinforced Clostridial Medium (RCM) (Thermo Scientific), and *Lactobacillus murinus* strain 21–100 (isolated from P23 NOD mouse cecal contents in Blaser Lab) were cultured in De Man, Rogosa, and Sharpe (MRS) medium (Thermo Scientific).

### Sample 16S microbiome analysis and statistical methods.

Fecal microbiota DNA from 18 subjects was extracted using the DNeasy PowerSoil-htp HTP 96 Kit (Qiagen, Hilden, Germany). The V4 region of bacterial 16S rRNA genes was amplified in triplicate reactions using barcoded fusion primers 515F/806R, which amplifies bacterial and archaeal 16S genes. The DNA concentration of the V4 amplicons for each sample was measured using the Quant-iT PicoGreen dsDNA assay kit (Life Technologies, Eugene OR). Samples were pooled in equal quantities. These set pools were then purified using the Qiaquick PCR purification kit (Qiagen) to remove primers, quantified using the high-sensitivity dsDNA assay kit and the Qubit 2.0 Fluorometer (Life Technologies) and then combined at equal concentrations to form the sequencing library. About 254 bp V4 region was sequenced using the Ilumina MiSeq 2×150bp platform. Quantitative insights for microbial ecology (QIIME, Version Qiime 2–2022.8) were used for quality filtering and downstream analysis for a-diversity (Shannon index), p-diversity (Jaccard distance matrix) and compositional analysis. Sequences were filtered for quality trimmed, de-noised, merged and then the chimeric sequences were removed using DADA2 plugin to generate the feature table. Taxonomy was assigned using Silva 138. Significant differences in alpha diversity between experimental groups were determined using Kruskal-Wallis method, while differences in p-diversity were tested by pairwise PERMANOVA with 999 permutations.

### Cell culture and incubation assays.

Murine macrophage NFkB-SEAP reporter RAW-Blue cells (InvivoGen Cells, cat# raw-sp, San Diego CA) were grown in Dulbecco Modified Eagle Medium (DMEM) (Corning, Tewksbury MA) supplemented with 4.5% glucose, 10% fetal calf serum (FCS) (Corning), 2 mM L-glutamine, 1 x Penicillin-streptomycin (Gibco, Waltham MA), and 100 µg/mL zeocin (InvivoGen Cells) in a humidified incubator with 5% CO2 at 37°C. The cells were passaged when they reached 70% confluence for 6–10 generations, then scraped, resuspended in fresh media, and plated in a flat-bottom 96-well tissue culture plate (Corning) at final density 2 × 10^5^ cells/well in 180 µL. The cells were incubated for 2 hours at 37°C in an atmosphere of 5% CO_2_, then treated with the target lipid compound at a final concentration from 0.1–125 µM in 1% (v/v) EtOH or with a blank reagent (EtOH) with final concentration 1% (v/v) as a control. After 1 hour of incubation, the lipid-cell co-cultures were treated with E *coii* LPS (Sigma -Aldrich) to a final concentration of 10 µg/mL. After 24 hours of stimulation, supernatants were collected, and NFkB activation determined using the detection medium QUANTI-Blue, prepared according to the manufacturer’s recommendations.

Human colonic epithelial cell line HT-29 (ATCC, Manassas VA) was cultured in RPMI 1640 medium (Corning) with 10% FCS and 1x penicillin-streptomycin in a humidified incubator with 5% CO2 at 37°C and passaged when they reached 90% confluence. For incubation with lipid compounds, the cells were scraped and resuspended in fresh media, and plated into 6-well tissue culture plates (Corning) and grown to >90% confluence in RPMI 1640 medium with 10% FBS without antibiotics (2 mL per well) and incubated for 1 hour to improve attachment, then each lipid compound added at a final concentration of 50 µM in 1% EtOH, or blank reagent EtOH as a reference control, incubating for 16 hours at 37°C in 5% CO2. The attached cells were washed with ice-cold PBS, resuspended in TRIzol reagent (Qiagen), and frozen at −80°C for RNA extraction.

To isolate mouse small intestinal epithelial cells (SIEC), male NOD mice were sacrificed at P23, the small intestine was removed and opened longitudinally, rinsed with cold HBSS (Gibco) to remove contents, and cut into 2-cm pieces, and incubated on ice in 20 mL DMEM (Corning) supplemented with 10% FCS (Gibco) with Collagenase type I (Sigma-Aldrich) 200 U/mL at 37°C for 30 min, with gently shaking at 100 rpm. The epithelial layer was gently dissociated by pipetting up and down from intestinal tissue and collected, filtered through a 100 µm cell strainer (Fisher Sci, Waltham MA) and centrifuged at 300 g for 5 min to pellet cells. Cells pooled from 3 mice were cultured and passaged in DMEM medium (Corning) supplemented with 10% FCS and 1x penicillin-streptomycin. For incubation with lipid compounds, fresh sub-cultured cells were seeded at high density into 12-well tissue culture plates (Corning) in DMEM medium with 10% FBS without antibiotics (1 mL per well), cultured to >90% confluence for 1 hour, then incubated in a 2 mL co-culture system for 16 hours at 37°C in 5% CO2 with each lipid compound (4 µL) at a final concentration of 50 µM in EtOH, or blank reagent EtOH as a reference control. The attached SIEC cells were washed with ice-cold PBS, resuspended in TRIzol and frozen at −80°C for RNA extraction.

### RT-qPCR for host target gene expression.

Total RNA was extracted from TRIzol-collected cells using the QIAgen Mini RNeasy kit (Qiagen) and cDNA was synthesized from the total RNA samples using the Verso cDNA kit (Thermo Scientific) according to the manufacturers’ instructions. qPCR was performed on a LightCycler 480 system (Roche, Branchburg NJ) using 10 ng of synthesized cDNA, target gene-specific primer pairs (Table S8), and Power SYBR Green PCR Master mix (Roche). Target mRNA levels were normalized to 18S rRNA or the housekeeping gene GAPDH as internal controls for each sample. For group mean comparisons, the Mann-Whitney t-test was performed, with p-value < 0.05 indicating significance.

### Mitochondrial respiration measurement.

The oxygen consumption rate (OCR) was measured using the Seahorse XF Cell Mito Stress Test Kit and the Seahorse XF Analyzer (Seahorse Bioscience, Location, USA), following the manufacturer’s instructions. Briefly, fresh HT29 cells were seeded in XF96 microplates at a density of 1.25 × 10^5^ cells/mL per well in 180 µL of DMEM (Corning) supplemented with 10% FBS (Corning) and 10 mM glucose and incubated for 24 hours at 37°C in a humidified atmosphere containing 5% CO_2_. Cells were then treated for 24 h with the target lipid compound LPG(16:0) at a final concentration of 0.5, 2, and 5 µM, or with LPG(18:0) at 0.5, 1, and 2 µM, in 0.25% (v/v) PBS. A PBS-only treatment (0.25% v/v) served as the vehicle control. Prior to the assay, the Seahorse XF sensor cartridge was hydrated with XF calibrant (200 µL/well) and incubated overnight in a humidified, non-CO_2_ 37°C incubator. The assay medium was prepared using XF DMEM (pH 7.4; Seahorse Bioscience) supplemented with 1 mM pyruvate, 2 mM glutamine, and 10 mM glucose. Cells were incubated and washed three times with assay medium. Three compounds were sequentially injected into the microplate: oligomycin (1.0 µM) via Port A, carbonyl cyanide-4-(trifluoromethoxy) phenylhydrazone (FCCP; 2.0 µM) via Port B, and a mixture of rotenone and antimycin A (0.5 µM each) via Port C, in accordance with the XF Cell Mito Stress Test protocol^32^. After the assay, OCR values were normalized to total protein content per well, measured using the BCA assay, to account for differences in cell number. Protein-normalized values were used to calculate basal mitochondrial respiration, maximal respiration, ATP-linked oxygen consumption, and non-mitochondrial respiration.

### Mitochondria and lysosome staining.

HT-29 cells were cultured in DMEM medium supplemented with 10% FBS on an ibiTreat µ-Slide 8-well chambered coverslip (Ibidi GmbH, Grafelfing, Germany) for 24 h and then treated with 5 µM LPG(16:0), 5 µM LPG(18:0), or blank PBS for 24 h. Following treatment, the medium was replaced with DMEM containing 2% FBS, and cells were incubated overnight with 15 nM tetramethylrhodamine Methyl Ester Perchlorate (TMRM; Fisher Scientific) to assess mitochondrial membrane potential. The next day, the medium was refreshed with DMEM containing 2% FBS, and cells further stained with 100 nM MitoTracker Green FM (Cell Signaling Technology, Danvers MA) and 25 µM LysoTracker Deep Red (Thermo Fisher Scientific) for 4 h to label total mitochondrial mass and lysosomes, respectively^33^. After staining, cells were washed with PBS and immediately imaged using a Zeiss LSM 900 confocal microscope. Quantitation of total mitochondrial mass and lysosomal content was performed using Zeiss LSM software and ImageJ software. Statistical analysis was based on measurements from 20 cells using Student’s t-test.

### Oral administration of lipids to mice.

To evaluate the *in vivo* effects of administering specific lipid compounds to young NOD mice, dams and their litters were randomly assigned to control (C) or 1PAT antibiotic (1P) groups as previously described^15^. A therapeutic dose of the macrolide tylosin tartrate (Sigma-Aldrich, Billerica MA) was given to 1P pups in their non-acidified drinking water at 333 mg/L on P5-P10. At postnatal day (P) 12, pups from 3 litters in the 1P group received a single gavage of fresh prepared lipid compounds LPG(13:0), PG(15:0_15:0), LPG(16:0), or LPG(18:0), respectively, at a dose of 3 mg/kg body weight (60 µL) daily for one week to P18. As controls, pups in the C group received the same volume of the blank solvent reagent (PBS-2% EtOH). Additional litters of 1P mice received: (i) cecal material transfer (CMT) from a pool of cecal contents from 3 male NOD mice at P23; or (ii) retinoic acid (3 mg/kg body weight) in a single daily dose from P12 to P18. From each litter, four pups (2 male/2 female) were sacrificed on P19, and ileum and colon collected in RNAlater for RT-qPCR-based evaluation of expression of host genes associated with T1D development. Cecal contents were also collected and frozen for lipidomic analysis and 16S rRNA gene sequencing. To compare the in vivo effects of specific lipid compounds on immune profiles using spectral flow cytometry, 3-week-old male C57BL/6N mice were obtained from Taconic Farms Inc. mice were randomly assigned to control (C) or 1PAT antibiotic (1P) groups. Mice in the 1P group received a therapeutic dose of the macrolide tylosin tartrate (Sigma-Aldrich) as above from P28 to P33. Beginning at P33, 1P mice were administered lipid compounds LPG(16:0), LPG(18:0), or PBS only by oral gavage as above for one week. Control mice received PBS only by gavage following the same schedule. All mice were euthanized at P40 for collection of spleen and mesenteric lymph nodes (MLNs) for spectral flow cytometry analysis, and cecal contents for microbiome profiling.

### DNA extraction and microbiome sequencing.

Total DNA was extracted from cecal, and ileal samples using the DNeasy PowerSoil HTP 96 Kit (QIAGEN, Valencia CA) following the manufacturer’s instructions. An amplicon library of the bacterial 16S rRNA V4 regions was obtained using barcoded fusion primers 515F/806R. The DNA concentration of each amplicon was quantified using the Quant-iT PicoGreen dsDNA Assay Kit (Invitrogen) and the SpectraMax iD3 microplate reader (Molecular Devices, San Jose CA). Samples were pooled and purified with the QIAquick PCR Purification Kit (QIAGEN), and the pooled samples were quantified using the Quant-iT dsDNA Assay Kit, high sensitivity (Invitrogen) on a Qubit 2.0 Fluorometer (Life Technologies, Carlsbad CA) and then combined at equimolar concentrations to form the sequencing library. Paired-end sequencing (2×150bp) of the constructed library was subsequently performed on the Illumina MiSeq platform (Illumina, San Diego CA) at Azenta Life Sciences (South Plainfield NJ).

### Bioinformatics analysis of microbiome sequences.

Raw paired end reads with perfect matching of bases between forward and reverse sequences were retained, demultiplexed, filtered, and analyzed using the QIIME 2 v2024.10 pipeline as described^34^. Briefly, the identification of OTUs was performed using the DADA2 plugin with quality filtering to trim the first six bases of each sequence with quality score < 35. Taxonomy was assigned to sequences using the Naive Bayes classifier compared against a SILVA 138 99% similarity OTUs reference database trained on the 515F/806R region of the 16S rRNA gene^35,36^. Ileal samples were rarefied at 4,000 reads, and cecal samples were rarefied at 11,000 reads for assessing microbial a- and p-diversity differences. To evaluate species richness and evenness, a-diversity was computed by two commonly used metrics: phylogenetic diversity (Fatith’s PD), and Pielou’s evenness, respectively. In addition, the Shannon’s index was used to estimate both richness and evenness in a single equation. To assess the overall microbial community variation between samples, Bray-Curtis matrices were used for p-diversity analysis. The multivariable association analysis with linear models (MaAsLin 2) was implemented in R to detect significant differences in relative abundances of microbial taxa between study groups^37^. Visualizations were created in R with data extracted from QIIME 2 artifacts using qiime2R (v0.99.6)^38^.

### Statistical analysis.

Measurements of microbial a-diversity and the relative abundance of bacterial communities and species were analyzed using the GraphPad Prism 9 software and displayed as mean ± standard error of mean in scatter plots, box-and-whiskers ± min-to-max, or heatmaps. Comparisons between study groups were assessed Kruskal-Wallis run sum test to account for additional variables. Statistical significance of inter- and intra-group p-diversity was determined by a permutational multivariate analysis of variance (PERMANOVA). Principal coordinate analysis (PCoA) was applied to create ordinations and to visualize the diversity between samples. A Benjamini-Hochberg-corrected q value < 0.25 was used by MaAsLin2 to determine statistical significance. A *p* value < 0.05 was considered statistically significant for all other statistical analyses throughout this study.

### RNA-seq and bioinformatics analysis.

Total RNA was extracted from mouse ileal tissues using the RNeasy Plus Mini Kit (Qiagen) combined with on-column DNA digestion using the RNase-Free DNase Set (Qiagen). RNA quality and quantity were determined using NanoDrop (NanoDrop Technologies). Libraries were prepared with rRNA depletion method. Subsequent sequencing was performed on Illumina platform (30 million paired-end reads per sample) with ERCC spike-in at Azenta Life Sciences (South Plainfield, NJ). RNA-Seq data generated for were deposited in the NCBI Gene Expression Omnibus (https://www.ncbi.nlm.nih.gov/geo/). Raw sequencing reads (fastq) were quality checked using fastQC (v.0.12.1) ^39^ and aligned to mouse mm9 reference genome using Kallisto (v2.1.0)^40^. Differential expression analysis was performed with DESeq2 (v.1.46.0; https://bioconductor.org/packages/DESeq2)^41^ and KEGG pathways analyses was performed with enrichKEGG package in the R interface^42^. All volcano plots were generated with the EnhancedVolcano package in the R interface^43^. All heatmaps were generated with the pheatmap package^44^ using as the Euclidean distance metric for non-supervised hierarchical clustering.

### Spectral flow cytometry.

From each mouse, mesenteric lymph nodes (MLNs) and spleen were collected and stored on ice in RPMI 1640 medium supplemented with 10% fetal bovine serum and 1% penicillin-streptomycin (RPMI/FBS/PS) after sacrifice. Spleens were dissociated through a 70 µm cell strainer using a 1 mL syringe plunger. The strainer was flushed with RPMI/FBS containing collagenase D (1 mg/mL) and DNase I (0.5 mg/mL), and cells were incubated for 15 min at 37 °C. Following filtration through a fresh 70 µm strainer, cells were centrifuged and red blood cells lysed with 1 mL 1X RBC lysis buffer (eBioscience, San Diego CA) for 3 min at room temperature. Lysis was stopped with PBS/BSA, and cells were washed and resuspended in 2 mL eBioscience^™^ Flow Cytometry Staining Buffer. 100 µL was aliquoted for staining. MLNs were dissociated through a 40 µm strainer using a 1 mL syringe plunger and washed with PBS/BSA. Cells were centrifuged, resuspended in 100 µL Flow Cytometry Staining Buffer (eBioscience), and reserved for staining. Spleen and MLN cells were transferred to a 96-well U-bottom plate and blocked with anti-CD16/CD32 (Fc block; BioLegend, 1:100 in 1X PBS, Corning) for 20 min on ice. A 25 µL antibody master mix containing extracellular markers and viability dye in Brilliant Stain Buffer (Thermo Fisher) was added (Supplementary Table 7), and cells were incubated for 20 min on ice. Cells were washed with 100 µL eBioscience^™^ Flow Cytometry Staining Buffer and centrifuged, then fixed with 50 µL of Fixation/Permeabilization Concentrate and Diluent (Foxp3/Transcription Factor Staining Buffer Set, eBioscience) according to the manufacturer’s instructions, washed with 10X diluted Permeabilization Buffer (PB), and stored overnight at 4 °C in 50 µL PB. The next day, cells were washed with PB and stained with the intracellular antibody master mix (Supplementary Table 7), in the same buffer for 50 min. After washing, cells were resuspended in 200 µL Flow Cytometry Staining Buffer for acquisition. Single-stained controls were prepared using splenocytes and Ultracomp eBeads^™^ Plus (Thermo Fisher). Live/dead controls were generated using splenocytes, MLN cells, or the ArC^™^ Amine Reactive Compensation Bead Kit. Fluorescence-minus-one (FMO) controls were prepared using splenocytes. Data were acquired on a 4-laser Cytek^®^ Aurora (Cytek Biosciences). Unmixing was performed on the Cytek^®^ Aurora using single-stained controls from splenocytes and beads. Manual gating was carried out on unmixed .fcs files using FlowJo v10 (FlowJo/BD), and subsequent analysis followed the MARMOT workflow^45^.

## Supplementary Material

Supplement 1
