# Supplementary material for: Gut microbiota phospholipids regulate intestinal gene expression and can counteract the effects of antibiotic treatment
Source: Res Sq. 2025 Dec 2:rs.3.rs-7924457. Preprint. [Version 1] doi: 10.21203/rs.3.rs-7924457/v1 (PMC12687810; doi:10.21203/rs.3.rs-7924457/v1)
Supplement: Supplement 1 [file NIHPPrs7924457v1-supplement-1.pdf]

Supplementary Files

This is a list of supplementary files associated with this preprint. Click to download.

- TableS1.xlsx
- TableS5.xlsx
- TableS4.xlsx
- NatureLettersSupplementalmaterials.docx
